# Minimally invasive prone transpsoas lateral lumbar interbody fusion using navigation

**DOI:** 10.3171/2026.4.FOCVID2639

**Published:** 2026-07-01

**Authors:** Shailen G. Sampath, Alex Hernandez Manriquez, Andrew S. Zhang, Nathan J. Winans, Vivian P. Le, Dean Chou, Paul Park, Andrew K. Chan

**Affiliations:** 1 Department of Neurological Surgery, Columbia University, New York;; 2 Department of Orthopaedic Surgery, Columbia University, New York, New York; and; 3 Department of Neurosurgery, Semmes Murphey Clinic, Memphis, Tennessee

**Keywords:** PTP, prone transpsoas approach, Stealth, minimally invasive, lateral lumbar interbody fusion, navigation

## Abstract

Minimally invasive spine surgery continues to evolve, with the single-position prone transpsoas lateral approach offering simultaneous lateral and posterior access without repositioning. This technique minimizes tissue disruption while enabling efficient single-position surgery. The authors present the case of a 57-year-old male with left-sided back pain and radiculopathy recalcitrant to conservative treatment. MRI revealed multilevel degeneration, with single photon emission CT (SPECT) highlighting L2–3 pathology. After exhausting conservative measures, he underwent a minimally invasive prone lateral lumbar interbody fusion at L2–3 with the use of navigation. This video highlights the technical steps and advantages of this approach and navigated technique.

The video can be found here: https://stream.cadmore.media/r10.3171/2026.4.FOCVID2639

## Transcript

My name is Andrew Chan, and in this video I highlight the use of navigation during a minimally invasive prone lateral lumbar interbody fusion performed via a transpsoas approach.^1^ Please note consent was obtained from the patient for this video.

### 0:32 Clinical Presentation.

The patient is a 57-year-old man who presented with 18 months of left-sided low back pain and left anterior thigh and knee pain and paresthesias refractory to conservative management. He was neurologically intact.

### 0:42 Imaging.

EOS imaging demonstrated no significant sagittal or coronal malalignment. Lumbosacral x-rays showed degenerative changes without gross instability on dynamic views.

T2-weighted MRI revealed multilevel degenerative disc disease with a left foraminal disc protrusion at L2–3, along with left lateral recess stenosis. Modic changes were also seen within the endplates.

CT imaging showed vacuum disc phenomenon and asymmetric disc space collapse on the left at L2–3. CT-SPECT imaging revealed focal increased signal intensity at L2–3, further supporting this as the primary symptomatic level given this was ipsilateral to his left-sided back pain.

### 1:15 Management.

A left L2–3 transforaminal epidural injection provided only temporary relief. Given persistent axial back pain and radiculopathy, a navigated L2–3 prone lateral interbody fusion was pursued.

### 1:26 Operative Room Setup and Patient Positioning.

The operating room was equipped with an O-arm for intraoperative CT-based imaging, a 3D-navigation system, C-arm fluoroscopy, a 4K retractor-based camera, and neuromonitoring.

The patient was positioned prone on a radiolucent table with the arms in a comfortably abducted position, with chest, hip, and thigh pads. Proprietary pelvic and thoracic bolsters were used to permit coronal bending, with bolsters placed near the greater trochanter and caudal to the axillae.

An AP fluoroscopic image was obtained to ensure a true AP position of the L2–3 segment. A lateral fluoroscopic image was obtained to mark the disc space and ensure bolster placement did not obstruct the lateral approach to L2–3. Sterile draping was then performed, making sure to use a wide enough drape to include the left lateral incision, the percutaneous screw incisions, and the iliac navigation pin.

### 2:07 Percutaneous Pedicle Screw Placement With Navigation.

Percutaneous pedicle screws were placed under navigation. A reference array was placed into the left posterior superior iliac spine via an in-out-in technique, on the side ipsilateral to the prone lateral approach. Ipsilateral placement is important to allow the camera to visualize both the reference array and navigated instruments in the same field.

The intraoperative CT was then brought in to acquire imaging for navigation. The navigation system was used to register the instrumentation. Once registered, the navigation wand was used to identify starting points and trajectories of the intended pedicle screws. Starting points were marked on the skin and bilateral incisions were created to accommodate pedicle screw towers. Typically, a 1-inch incision is sufficient for a single-segment surgery.

A navigated drill guide was used to cannulate the pedicle, followed by guidewire placement into the hole. The navigated drill guide was then removed over the guidewire, with the wire firmly secured in place. An undersized cannulated and navigated tap was then placed over the guidewire, and the tap was advanced with navigation.

After tapping and removing over the guidewire, appropriately sized screws were advanced over the guidewires under navigation. The guidewire was then removed.

### 3:03 Prone Transpsoas Lateral Lumbar Interbody Fusion.

Once all pedicle screws were placed, a coronal bend was introduced by adjusting the bed bolsters. This step may not be necessary in all patients, but it does often enhance access to the upper lumbar or L4–5 levels by increasing the window between the iliac crest and the ribs.

After the coronal bend is induced, an intraoperative CT was repeated to confirm screw placement and to update the navigation for the lateral approach. It is important that this scan is performed eccentric to the side of the lateral approach to capture the relevant anatomy.

First, facet decortication was performed. This step not only facilitates facet arthrodesis, but it also importantly loosens up the segment to accommodate cage placement. A navigated burr was used to decorticate the L2–3 facet joints bilaterally, followed by packing them with bone graft with navigated dilators via a plunger technique. Use of navigation for this step enables the surgeon to ensure thorough decortication and sufficient disruption of the joint.

For the prone lateral portion, the OR is set up as follows: the surgeon will be on the left of the patient. A table-mounted retractor is cranial to the patient’s incision. The reference array remains in left PSIS. The scrub tech is on the same side as the surgeon. The optical camera is positioned at the foot of the bed, ipsilateral to the approach, to maintain line of sight with the array and instruments. The navigation, fluoro, and retractor camera monitors are opposite the surgeon. Of note, the Mayo stand is also to the left of the patient, and lowered to avoid blocking the optical camera.

In practice, the surgeon is positioned to see the screens for the navigation system, the retractor-mounted camera, and the C-arm while comfortably seated. While some surgeons opt to tilt the bed, it is my preference not to in order to avoid working at nonorthogonal trajectories.

After setup, the navigation wand was used to plan the incision. A point parallel to the PLL and a point parallel to the ALL at the index level are marked, and the incision was planned incorporating a line between these points. This should roughly correspond with the skin marking from the beginning of the case.

Following planning, a skin incision was made, followed by blunt dissection through the external oblique, internal oblique, transversus abdominis muscles, along with the transversalis fascia, to access the retroperitoneal space. Finger dissection developed the retroperitoneal plane permitting unhindered access to the psoas muscle. A safe corridor involves first sweeping dorsally to identify the quadratus lumborum, and then sweeping deep and ventrally to identify the bulbous psoas. Adherence to this technique ensures that visceral structures are mobilized ventrally, away from the lateral approach.

The triggered EMG is connected to the navigated dilator and the stimulation is turned on. A finger was kept in place on the psoas—as a shield against the viscera—and the navigated probe was passed dorsally over the finger.

Navigation was then used to find the ventral portion of the disc, in the ventral portion of the psoas, and then moved back slowly to the midpoint of the disc. Not typically an issue at L2–3, but if low stimulation thresholds are encountered, the navigated probe may be used to efficiently find a new trajectory while triggered EMG is live. This permits real-time safe trajectory adjustment, while reducing fluoroscopic exposure. Navigation also enhances avoidance of critical structures such as vascular structures, the spinal canal, the anterior longitudinal ligament, or bowel. With navigation, we are also able to confirm the length that will be needed for the final cage by using the projection line on the dilator as a caliper.

Here we take a moment to briefly show another case for a patient who underwent surgery at L4–5 for grade 2 spondylolisthesis to highlight how navigation permits simultaneous advancement of the dilator while monitoring with triggered EMG. Initially, stimulation thresholds were somewhat low. However, with navigated repositioning of the dilator, a new trajectory was efficiently found with an acceptable higher stimulation threshold, before advancing the K-wire. This stands in contrast to fluoroscopic-based approaches where a number of lateral images are needed when repositioning the initial dilator.

Back to the present case, sequential dilators were advanced and stimulated. The retractor was advanced over the largest dilator. The navigation array was then reattached to the initial dilator to ensure orthogonal placement of the retractor before locking the position with the table-mounted retractor arm.

The retractor was then opened to visualize the disc space. The navigation wand was placed along the ventral and dorsal blades to ensure safe placement of the retractor and avoidance of the ALL and spinal canal.

The dorsal blade was stimulated to confirm no low stimulation thresholds, and a dorsal shim was inserted to anchor the retractor to the spine, thereby preventing migration during disc preparation.

### 7:13 Visualizing the Disc Space.

A light was then inserted into the ventral blade and the retractor-based camera was inserted into the dorsal blade.

First, we coagulated the few traversing psoas fibers with bipolar cautery to have a clear visualization of the disc space.

A rectangular annulotomy was performed with a disc scalpel and then a navigated Cobb was advanced down the middle of the disc space. Navigation here ensures the endplate is not violated and that the contralateral annulus is released. With navigation, we do not ride the two endplates with the Cobb as even a small inaccuracy of the navigation may risk endplate violation.

A pituitary was then used to remove disc material.

Other standard disc prep instruments can be navigated as well. Here, we are using a navigated ring curette to remove more disc material, including the cartilaginous endplate, and to ensure we do not extend past the confines of the disc space.

After a thorough discectomy with the navigated instruments, a navigated trial was advanced. Sequentially upsized trials were then also advanced until the desired cage height and lordosis was achieved. Navigation offers a synthetic AP and lateral view, if the surgeon desires a radiograph-type view to visualize the location of the trial and ultimate cage placement.

### 8:11 Final Implant Placement Without Navigation.

The C-arm was brought in for final implant placement as this step is not navigable. The implant was then inserted with fluoroscopic assistance. The retractor was removed and hemostasis achieved.

### 8:20 Percutaneous Rod Placement and Closure.

The coronal bend was then removed from the bed before rod placement. Calipers were used to confirm the appropriate length of the rods. The rods were passed with rod handles. Set screws were final tightened and the rod handles and the percutaneous towers were removed.

The C-arm was used to obtain final AP and lateral imaging.

A multilayer closure was performed for all incisions.

### 8:38 Postoperative Course.

Postoperatively, the patient was admitted to the floor and his pain was managed with oral medications. The patient was safely discharged on postoperative day 1 without complications. Postoperative lumbar and standing EOS films demonstrated satisfactory position of the instrumentation.

At 3 months, the patient was doing very well, with resolved leg and back pain, and no complications or symptoms related to lateral exposure. Our hope is that through this case we were able to highlight the benefit of using navigation for prone lateral lumbar interbody fusion.

This slide summarizes the benefits of navigated prone lateral lumbar interbody fusion. First, it helps to facilitate efficient and precise placement of the dilator and retractor with no or minimal fluoroscopic shots. Secondly, navigation provides a method to easily reposition the dilator while triggered EMG is running. This may be particularly helpful for L4–5, large osteophytes, obese patients, and a high-riding crest or obstructing rib. Importantly, it helps to reduce radiation exposure to the surgeon and staff.

### 9:28 Advantages of Navigated Prone LLIF Compared to Traditional Lateral Decubitus LLIF.

It may also be helpful to highlight some key differences between the navigated prone transpsoas approach compared to the traditional lateral decubitus approach for lateral lumbar interbody fusion.^2,3^ The prone approach optimizes lordosis and improves the lateral working corridor, as the psoas and lumbar plexus shift dorsally while the peritoneal contents descend ventrally in the prone position.^4,5^ In addition, this approach allows for single-position surgery, enabling both the lateral interbody fusion and posterior instrumentation to be performed without repositioning. As a result, operative efficiency may be improved by avoiding the need for repositioning and by permitting posterior work to be performed concurrently if desired. Prone positioning may also be particularly helpful in more challenging anatomy. For example, passive spondylolisthesis reduction from positioning alone, or the ability to perform posterior work first, such as with facetectomies with screw-and-rod instrumentation with active spondylolisthesis reduction, may help facilitate a safer lateral working corridor in challenging cases, including those with a ventrally positioned psoas or plexus or those with a grade 2 L4–5 spondylolisthesis. Finally, 3D image acquisition and navigation are employed in the more familiar and stable prone position, which may further streamline workflow for surgeons accustomed to posterior spinal procedures.

### 10:32 Special Considerations.

There are also considerations that merit specific discussion. First, the distance from the skin to the spine may be longer relative to the lateral decubitus position. As a result, in obese patients, a wider incision may sometimes be required to permit safe blunt retroperitoneal dissection to the psoas, and longer retractor blades may also be necessary. In addition, surgeons who are more familiar with the lateral decubitus approach may encounter a learning curve when adopting the prone transpsoas technique. Finally, the table-breaking capacity may be lower compared with the lateral decubitus position, so angled instruments may be more frequently required.

## Disclosures

A. Zhang reported personal fees from OSSDsign outside the submitted work. D. Chou reported personal fees from Globus and Medtronic outside the submitted work. P. Park reported royalties from Globus; personal fees from Globus, Medtronic, Cerapedics, and Kuros; stock in ATEC; and grants from Cerapedics, ISSG, and SMISS outside the submitted work. A. Chan reported personal fees from Alphatec Spine, Carlsmed, and Spineart; and grants from Cervical Spine Research Society outside the submitted work.

## Author Contributions

Primary surgeon: Chan. Assistant surgeon: Zhang. Editing and drafting the video and abstract: Chan, Sampath, Hernandez Manriquez, Zhang, Winans. Critically revising the work: Chan, Sampath, Hernandez Manriquez, Zhang, Winans. Reviewed submitted version of the work: all authors. Approved the final version of the work on behalf of all authors: Chan, Le, Park. Supervision: Chan.

## Supplemental Information

### Patient Informed Consent

The necessary patient informed consent was obtained in this study.
